# Strongly polarizing weakly coupled ^13^C nuclear spins with optically pumped nitrogen-vacancy center

**DOI:** 10.1038/srep15847

**Published:** 2015-11-02

**Authors:** Ping Wang, Bao Liu, Wen Yang

**Affiliations:** 1 Hefei National Laboratory for Physical Sciences at the Microscale and Department of Modern Physics, University of Science and Technology of China, Hefei, Anhui 230026, China; 2 Beijing Computational Science Research Center, Beijing 100094, China

## Abstract

Enhancing the polarization of nuclear spins surrounding the nitrogen-vacancy (NV) center in diamond has recently attracted widespread attention due to its various applications. Here we present an analytical formula that not only provides a clear physical picture for the recently observed polarization reversal of *strongly coupled*^13^C nuclei over a narrow range of magnetic field [H. J. Wang *et al.*, Nat. Commun. 4, 1940 (2013)], but also demonstrates the possibility to strongly polarize *weakly coupled*^13^C nuclei. This allows sensitive magnetic field control of the ^13^C nuclear spin polarization for NMR applications and significant suppression of the ^13^C nuclear spin noise to prolong the NV spin coherence time.

The atomic nuclear spins are central elements for NMR and magnetic resonance imaging^1^ and promising candidates for storing and manipulating long-lived quantum information^2^ due to their long coherence time. However, the tiny magnetic moment of the nuclear spins makes them completely random in thermal equilibrium, even in a strong magnetic field and at low temperature. This poses severe limitations on their applications. The dynamic nuclear polarization (DNP) technique can bypass this limitation by transferring the electron spin polarization to the nuclear spins via the hyperfine interaction (HFI), but efficient DNP is usually prohibited at room temperature.

An exception is the nitrogen-vacancy (NV) center^3^ in diamond, which has an optically polarizable spin-1 electronic ground state with a long coherence time^4^, allowing DNP at room temperature^5^,^6^. This prospect has attracted widespread interest due to its potential applications in room-temperature NMR, magnetic resonance imaging and magnetometry^7^,^8^, electron-nuclear hybrid quantum register^9^,^10^,^11^, and electron spin coherence protection by suppressing the nuclear spin noise^12^. In addition to the remarkable success in coherently driving spectrally resolved transitions to initialize, manipulate, and readout up to three strongly coupled nuclear spins^10^,^13^,^14^,^15^,^16^, there are intense activities aiming to enhance the polarization of many nuclear spins via dissipative spin transfer from the NV to the nuclear spins. To overcome the large energy mismatch for resonant spin transfer, various strategies have been explored, e.g., tuning the NV spin near the excited state level anticrossing^6^,^17^,^18^,^19^,^20^,^21^,^22^ or ground state level anticrossing (GSLAC)^5^,^23^,^24^, driving the NV-nuclear spins into Hartman-Hahn resonance^25^,^26^ or selectively driving certain spectrally resolved transitions between hyperfine-mixed states under optical illumination^27^,^28^. Successful polarization of bulk nuclear spins in diamond have dramatically enhanced the NMR signal by up to five orders of magnitudes^17^,^28^ and significantly prolonged the NV spin coherence time^25^,^26^. In particular, near NV excited state level anticrossing, almost complete polarization has been achieved for the *on-site*^15^N (or ^14^N) and the *strongly coupled*^13^C nuclei in the first shell of the vacancy^6^,^20^,^21^,^22^,^29^.

Recently, Wang *et al.*^24^ exploited the GSLAC to achieve near complete polarization of the strongly couled, first-shell ^13^C nuclei and observed multiple reversals of the polarization direction over a narrow range (a few mT) of magnetic field. This interesting observation allows sensitive control of the polarization of *strongly coupled*^13^C nuclei by tuning the magnetic field, but a clear physical picture remains absent. Furthermore, in most of the existing works, only a few *strongly coupled* nuclear spins (HFI 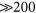 kHz) are significantly polarized via direct spin transfer from the NV center, while many weakly coupled nuclear spins are only slightly polarized via nuclear spin diffusion. Enhancing the polarization of these weakly coupled nuclear spins could further improve NMR and magnetic resonance imaging^17^,^27^,^28^ and prolong the NV spin coherence time^25^,^26^.

Motivated by the experimental observation of Wang *et al.*^24^, in this paper we present an analytical formula for the DNP induced by an optically pumped NV center near the GSLAC at ambient temperature. It not only provides a clear physical picture for the experimentally observed polarization reversal of the *strongly coupled*^13^C nuclei (with HFI ~ 100 MHz) over a narrow range (a few mT) of magnetic field^24^, but also reveals a simple strategy to (i) strongly polarize *weakly coupled*^13^C nuclei (with HFI down to ~kHz) and (ii) control the direction of their polarization by tuning the magnetic field over a much narrower range (~0.1 mT). First, we introduce an intuitive physical picture for our strategy. Then we present an analytical formula that substantiates this physical picture. Finally, we perform numerical simulations that demonstrate our strategy for a few hundred weakly coupled ^13^C nuclei.

## Results

### Model and intuitive physical picture

Our model consists of a negatively charged NV center coupled to many surrounding nuclear spins at ambient temperature. The NV center has a ground state triplet 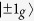 and 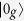 separated by zero-field splitting 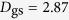 GHz and an excited state triplet 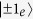 and 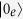 separated by zero-field splitting 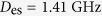
29. In a magnetic field *B* along the N-V axis (*z* axis), the electron Zeeman splitting 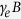 cancels the ground state zero-field splitting at a critical magnetic field 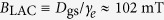, leading to GSLAC between 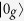 and 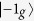. The GSLAC reduces the energy mismatch for the NV-nuclei flip flop and enables NV-induced DNP through their HFI 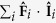, where 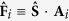 and 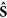 is the NV ground state spin.

Now we provide an intuitive physical picture for using an optically pumped NV center near the GSLAC to strongly polarize the nuclear spins and control their polarization direction by the magnetic field. For brevity we focus on one ^13^C nuclear spin-1/2 and drop the nuclear spin index *i*. Since 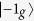 is nearly degenerate with the NV steady state 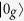 under optical pumping, the NV-nucleus flip flop is dominated by the nuclear spin raising transition 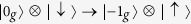 and the nuclear spin lowering transition 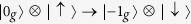, where 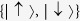 are nuclear spin Zeeman eigenstates. The energy mismatch of the raising (lowering) transition is 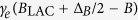 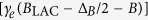, where 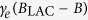 is the 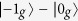 energy separation and 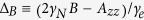 comes from the nuclear Zeeman term 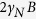 and the longitudinal HFI term 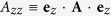. Since we always work near the GSLAC, where 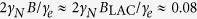 mT, we can regard Δ_Β_ as a constant independent of the magnetic field *B*. The different energy mismatches make it possible to *selectively* drive one transition into resonance while keeping the other transition off resonance: set the magnetic field to 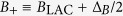 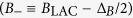, so the raising (lowering) transition has a vanishing energy mismatch, i.e., on resonance, while the lowering (raising) transition has a finite energy mismatch 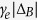, i.e., off resonance as long as the linewidth of the transition is smaller than 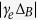. This highlights the linewidth (hereafter denoted by *R*) of the NV ground state as a crucial ingredient for achieving strong nuclear polarization: 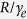 must be smaller than 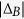, so that the raising and the lowering transitions can be spectrally resolved. Below we show that the optical pumping is the dominant level-broadening mechanism, so that 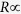 optical pumping strength. Therefore, strong negative (positive) nuclear polarization can be achieved under sufficiently weak optical pumping 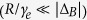 by tuning the magnetic field to *B*_−_ (*B*_+_). The direction of the polarization can be reversed by switching the magnetic field between *B*_−_ and *B*_+_. For first-shell ^13^C nuclei, 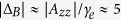 mT. This gives a simple explanation to the experimentally observed reversal of the polarization direction over a few mT^24^. For weakly coupled ^13^C nuclei, 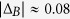 mT, so the direction of the polarization can be reversed by sweeping the magnetic field over a much smaller range. Below we substantiate this physical picture with an analytical formula.

### DNP theory of single nuclear spin

**Figure 1:**
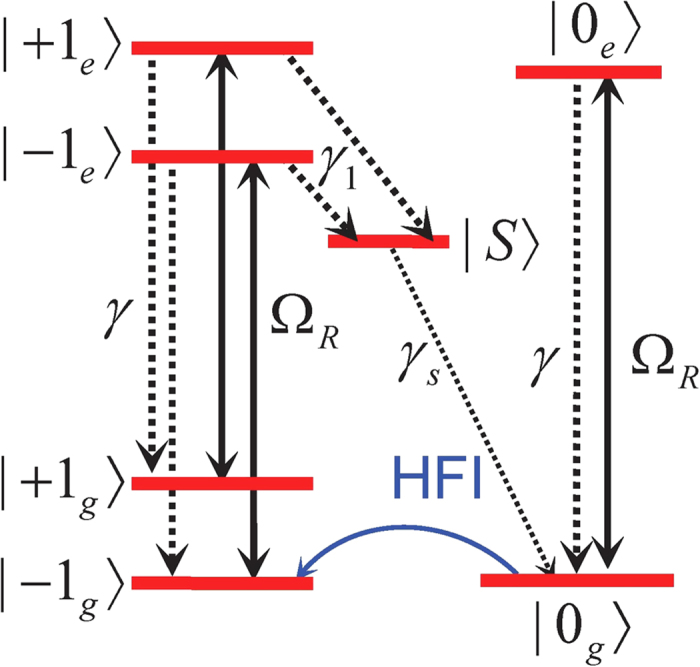
NV states at ambient temperature responsible for DNP under optical pumping.

Under optical pumping, seven energy levels of the NV center are relevant (Fig. 1). The NV-nucleus coupled system obeys the Lindblad master equation


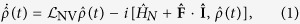


where 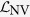 is the Liouville superoperator governing the NV evolution (including various dissipation processes as shown in Fig. 1) in the absence of the nuclear spin, 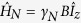 is the nuclear spin Zeeman Hamiltonian, and 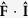 is the NV-nucleus HFI. Equation (1) can be solved exactly by numerical simulation. However, this approach does not provide a clear physical picture, and it quickly becomes infeasible for multiple nuclear spins (to be discussed shortly), because the dimension of the Liouville space grows exponentially with the number of nuclei.

Our work is based on a recently developed microscopic theory^30^,^31^,^32^. For the optically pumped NV center, this theory is applicable as long as the optical initialization time *τ*_*c*_ of the NV center is much shorter than the timescale of the DNP process, because in this case the NV center can be regarded as a non-equilibrium Markovian bath. Applying this theory to a ^13^C nuclear spin-1/2, we obtain the rate equation 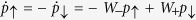 for the nuclear spin populations 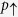 and 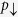 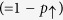, where *W*_+_(*W*_−_) is the rate of the nuclear spin raising (lowering) transition, as discussed in the previous subsection. The real-time evolution of the nuclear spin polarization 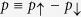 is given by 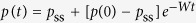, where 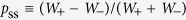 is the steady-state polarization and 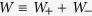 is the rate of DNP.

For weak optical pumping far from saturation, we can derive (see Methods) the following Fermi golden rule for the nuclear spin transition rates:


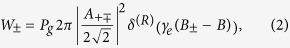


where 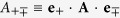 with 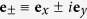, 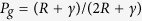 is the steady-state population of the NV center on 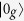, 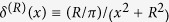 is the Lorentzian shape function, and the optically induced NV ground state level broadening *R is equal to the optical pumping rate*, i.e., the number of optical transitions per unit time from the NV ground orbital to the excited orbital (see Methods). This optically induced level broadening is typically much larger than the intrinsic NV spin decoherence rate (~1 kHz) and provides a microscopic explanation for the previously observed NV level broadening under laser illumination^23^,^33^. Most importantly, Eq. (2) quantifies the physical picture discussed in the previous subsection: to achive strong negative (positive) nuclear polarization, we can use weak optical pumping 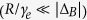 and set the magnetic field to *B*_−_ (*B*_+_), so that the rate *W*_−_ (*W*_+_) of the nuclear spin lowering (raising) transition is resonantly enhanced, while the rate *W*_+_ (*W*_−_) of the nuclear spin raising (lowering) transition is suppressed. The relation 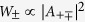 also suggests that the polarization *p*_ss_ depends strongly on the HFI tensor **A**, e.g., for the ^15^N nucleus with 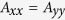 and 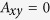, we have 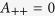 and 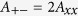, and hence 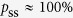.

Equation (2) is accurate only when the DNP time 1/*W* calculated from Eq. (2) 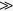 NV optical initialization time 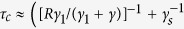, so that the NV center is a Markovian bath. When the optical pumping rate *R* is so small or the HFI is so strong that the DNP time calculated from Eq. (2) drops below *τ*_*c*_, the NV center becomes a non-Markovian bath and Eq. (2) becomes inaccurate, e.g., instead of dropping below *τ*_*c*_, the true DNP time would be lower bounded by ~*τ*_*c*_. This can be easily understood: since the nuclear spin dissipation originates from the dissipation of the optically pumped NV center, the timescale 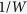 of the nuclear spin dissipation should be longer than the time sclae *τ*_*c*_ of the NV center dissipation.

Up to now, we have neglected other nuclear spin relaxation mechanisms, such as spin-lattice relaxation and HFI with NV excited states. The former occurs on the time scale ranging from a few seconds to tens of minutes^17^,^34^. The latter can be estimated from the Fermi golden rule, e.g., for a ^13^C located at 3Å away from the NV center, the dipolar HFI gives a nuclear spin relaxation time >1 s. These effects are described by nuclear depolarization 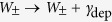, which increases *W* by 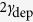 and decreases 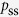 by a factor 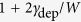, i.e., nuclear depolarization is negligible when 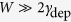.

Below we first specialize to strongly coupled ^13^C nuclei, where 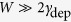 is always satisfied, so the nuclear depolarization is negligible. Then we consider weakly coupled ^13^C nuclei, where 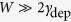 becomes a crucial condition for achieving strong nuclear polarization. In all our calculations, we use the experimentally measured parameters at room temperature (cf. Fig. 1): excited state orbital pure dephasing 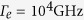
35, radiative decay rate *γ* = 13 MHz^36^, and intersystem crossing rates 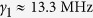
37 and 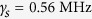
38,39,40,41. Unless explicitly mentioned, the very small leakage from 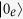 to 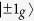 is neglected, consistent with the experimentally reported^14^,^42^ high optical initialization probability ~96% into 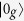. The effect of imperfect NV optical initialization will be discussed shortly.

### DNP of strongly coupled ^13^C nucleus

To begin with, we consider the DNP of a *strongly coupled*^13^C nucleus in the first shell of the NV center under optical pumping near the 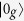
–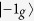 GSLAC. This configuration has been studied experimentally in ensembles of NV centers^24^, which shows a reversal of the direction of the ^13^C nuclear polarization over a narrow range (~a few mT) of magnetic fields. This interesting observation allows sensitive control of the polarization of the strongly coupled ^13^C nuclei by tuning the magnetic field, but a clear physical picture remains absent. Here the very strong HFI makes the DNP time calculated from Eq. (2) much shorter than 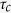, so our analytical formula are not accurate. In this case, we numerically solve Eq. (1) using the experimentally measured ground state HFI tensor^19^,^24^,^43^,^44^ with nonzero components 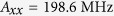, 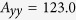 MHz, 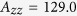 MHz and 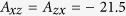 MHz. To focus on the intrinsic behavior of the DNP, we set 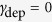. We have verified that due to the strong HFI induced NV-^13^C mixing, the ^13^C nuclear polarization depends weakly on the optical pumping up to 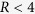 MHz. In our numerical calculation, we take 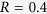 MHz.

**Figure 2:**
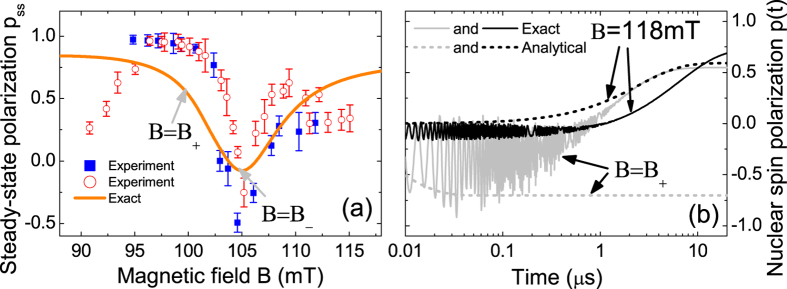
(**a**) *p*_ss_ of a first-shell ^13^C nucleus: exact numerical solution to Eq. (1) (orange solid line) compared with the experimentally deduced nuclear polarization^24^ (filled squares and empty circles) from two different analysis methods. (**b**) Real-time evolution of nuclear polarization 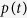 at *B*_+_ or far from *B*_+_: exact solution to Eq. (1) vs. our analytical formula Eq. (7).

In Fig. 2(a), the numerically calculated nuclear polarization (black solid line) correctly reproduces the sign reversal of the experimentally deduced nuclear polarization^24^ (circles and squares). Actually, although our analytical formula in Eq. (2) is not accurate, it still provides a clear physical picture for the polarization reversal: the negative polarization dip in Fig. 2(a) originates from the resonance of the nuclear spin lowering transition at 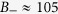 mT, while the positive polarization away from *B*_−_ originates from the dominance of the raising transition rate 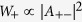 over the lowering transition rate 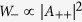 since 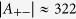 MHz is significantly larger than 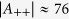 MHz. We notice that there is significant difference between our numerical results and the experimentally deduced nuclear polarization from the ODMR spectrum, especially in the magnitude of the negative dip. We tentatively attribute this discrepancy to two factors. First, all the parameters used in our calculation are taken from previous experimental measurements and/or first-principle calculations, which may differ from the particular experiment of Wang *et al.*^24^. Second, the estimate of the nuclear polarization from the ODMR spectrum involves a series of assumptions such as the lack of quantum coherence between different NV-^13^C mixed levels, so the uncertainty of this estimate is relatively large, e.g., the estimates based on different transitions give different results, denoted by the circles and squares in Fig. 2(a).

**Figure 3:**
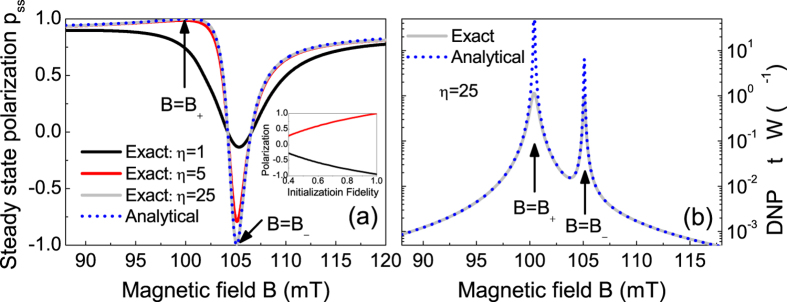
(**a**) Steady-state polarization *p*_ss_ of a first-shell ^13^C nucleus: exact numerical solution to Eq. (1) (solid lines) vs. analytical formula Eq. (2) (dotted line). To illustrate the convergence of our theory, we set 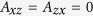 and reduce 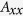 and 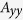 by a factor 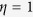 (black line), 5 (red line), and 25 (gray line). (**b**) DNP rate for 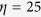: exact solution to Eq. (1) (gray solid line) vs. analytical formula Eq. (2) (blue dotted line).

For the first-shell ^13^C nucleus^24^, the NV center is not a Markovian bath and the numerically calculated nuclear polarization [solid lines in Fig. 2(b)] exhibit strong non-Markovian oscillation. In this regime, our analytical results [dashed lines in 2(b)] are not valid. However, as long as the DNP time 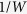 calculated from Eq. (2) is much longer than 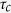, e.g., for weakly coupled ^13^C nuclei, our analytical formula becomes accurate. To demonstrate this point, we keep 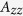 invariant, set 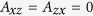, and reduce 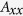 and 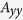 by a factor 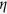 (i.e., 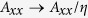 and 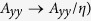, so that the nuclear spin transition rates 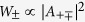 are reduced by a factor 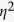. In Fig. 3(a), the agreement between the analytical results and the exact numerical results improves successively with increasing 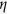, e.g., excellent agreement is reached for 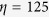. In Fig. 3(b), when the magnetic field approaches *B*_±_ (i.e., resonance of the raising or lowering transition), the DNP rate calculated from Eq. (2) sharply peaks and significantly exceeds 
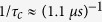
, while the exact numerical result saturates to ~(1.6 μs)^−1^. Away from *B*_±_, our analytical DNP rate 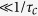 and hence agrees well with the exact result.

### DNP of weakly coupled ^13^C nucleus

An important feature in Fig. 2(a) is that when the HFI decreases, the positive (negative) polarization peak (dip) at *B*_+_ (*B*_−_) approaches +100% (−100%) due to the resonance of the nuclear spin raising (lowering) transition. This indicates the possibility of achieving strong positive (negative) nuclear polarization for weakly coupled nuclear spins by tuning the magnetic field to *B*_+_ (*B*_−_). In the above calculations, we have assumed perfect optical initialization of the NV center into 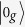 by neglecting the small intersystem crossing from 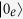 to 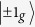. Actually, strong nuclear polarization can be achieved even when the NV initialization is not perfect: as shown in the inset of Fig. 3(a), the maximal nuclear polarization at *B*_±_ only decreases sublinearly with increasing initialization error, e.g., the nuclear polarization remains slightly above 80% when the optical initialization fidelity decrease to 80%. Since the nuclear polarization is not so sensitive to the NV optical initialization fidelity, hereafter we always assume perfect optical initialization of the NV center.

Now based on the analytical formula in Eq. (2), we discuss in detail the conditions for strongly polarizing distant ^13^C nuclei with weak HFI 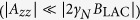, so that the separation between *B*_+_ and *B*_−_ is 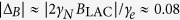 mT. Since we need to tune the magnetic field to *B*_+_ or *B*_−_, the experimental control precision *δB* of the magnetic field should be smaller than 0.08 mT. This is accessible by current experimental techniques, e.g., 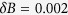 mT has been reported^34^. Another obvious condition is that the nuclear depolarization, which always tends to decrease the nuclear polarization, should be negligible, i.e., the maximal DNP rate  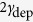 or equivalently 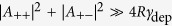. This requires that the HFI should not be too weak, e.g., for 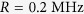 and 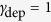 s^−1^, this requires the HFI to be larger than 1 kHz. Hereafter we assume that this condition is satisfied. The intuitive physical picture suggests that the linewidth *R* (=optical pumping rate) of the NV ground state is essential, so we divide our following discussion into strong optical pumping and weak optical pumping, respectively.

**Figure 4:**
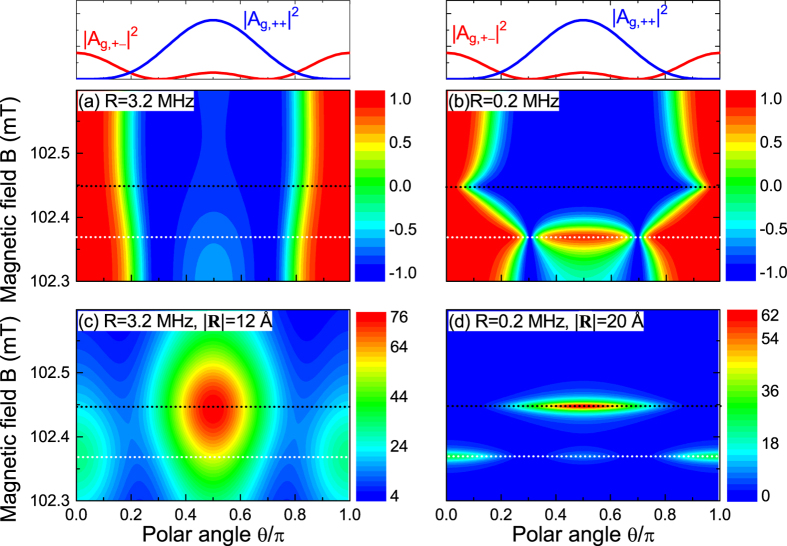
Steady state nuclear polarization *p*_ss_ [(**a**) and (**b**)] and DNP rate *W* in units of Hz [(**c**) and (**d**)] by dipolar HFI with the NV center, calculated from the analytical formula Eq. (2) with optical pumping rates *R* = 3.2 MHz [(**a**) and (**c**)] and 0.2 MHz [(**b**) and (**d**)].The distance of the ^13^C nucleus from the NV center is 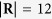 Å in (c) and 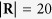 Å in (d). The white (black) dotted line indicates the magnetic field *B*_+_ (*B*_−_).

Under strong optical pumping 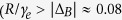 mT), the resonances at *B*_±_ are not spectrally resolved. In this case, the nuclear polarization is uniquely determined by the HFI tensor **A**. For dipolar HFI,


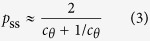


is uniquely determined by the polar angle *θ* of the ^13^C location **R** through 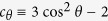. For example, in Fig. 4(c), the resonance of the nuclear spin raising transition at 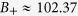 mT (marked by white dotted lines) and the resonance of the nuclear spin lowering transition at 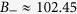 mT (marked by black dotted lines) are significantly broadened along the magnetic field axis and are not clearly resolved.

The most interesting case is weak optical pumping 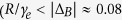 mT), where the nuclear spin raising and lowering transitions are spectrally resolved and can be selectively driven into resonance to achieve strong positive (negative) nuclear polarization by tuning the magnetic field to *B*_+_ (*B*_−_). For example, in Fig. 4(d), the very narrow resonance peak of the raising transition at *B*_+_ (marked by white dotted lines) and the resonance peak of the lowering transition at *B*_−_ (marked by black dotted lines) are clearly resolved. Correspondingly, in Fig. 4(b), these resonances give rise to strong positive (negative) nuclear polarization near *B*_+_ (*B*_−_), superimposed on the usual dependence on the polar angle *θ* via 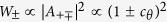 [plotted on top of Figs 4(a),(b)]. We notice that a ^13^C nucleus located at 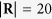 Å away from the NV center can be highly polarized at a rate ~60 Hz = (2.6 ms)^−1^, sufficient to overcome the slow nuclear depolarization 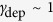 s^−1^.

### DNP of multiple ^13^C nuclei

Having established a complete picture for single-^13^C DNP, now we generalize the above results to many 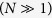 ^13^C nuclear spins coupled to the NV center via 
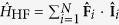
, where 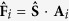 and the excited state HFI is neglected because the induced NV-nuclei flip-flop processes are off-resonant. In the same spirit as that in treating the DNP of a single nuclear spin, we decompose the HFI into the longitudinal part 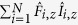 and the transverse part 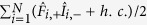. Then we approximate the longitudinal part with 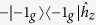 (which is treated *exactly*) and treat the transverse HFI with second-order perturbation theory, where


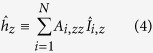


is the collective Overhauser field from all the nuclei.

The physical picture of the many-nuclei DNP is as follows. Up to leading order, the flip of different nuclei by the transverse HFI is independent, in the sense that at a given moment, only one nuclear spin is being flipped, while other nuclear spins simply act as “ spectators”. However, the flip of each individual nuclear spin does depend on the states of all the nuclear spins via the collective Overhauser field 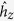: each many-nuclei state 
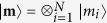
 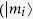 is the Zeeman eigenstate of the *i*th nucleus) produces an Overhauser field 
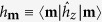
 that shifts the energy of the NV state 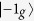 by an amount −*h*_**m**_. This renormalizes the 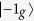
– 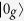 separation from 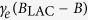 to 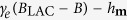 and hence changes the NV dynamics and NV-induced nuclear spin flip, e.g., the raising and lowering transition rates 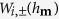 of the *i*th nucleus conditioned on the many-nuclei state being 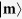 is obtained from Eq. (2) by replacing **A**, *B* with **A**_*i*_, 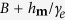, respectively.

The above physical picture is quantified by the following rate equations for the diagonal elements 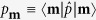 of the many-nuclei density matrix 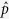:


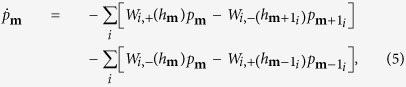


which has been derived by adiabatically decoupling the fast electron dynamics from the slow DNP process^30^,^31^,^32^. Here 
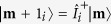
 is the same as 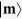 except that the state of the *i*th nucleus changes from 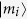 to 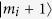. Now we discuss the difference between single-spin DNP and many-spin DNP. In the latter case, the DNP of each individual nucleus occurs in the presence of many “ spectator” nuclei, which produce a fluctuating Overhauser field 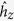 that randomly shifts the NV energy levels, such that the 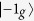
–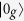 separation changes from 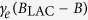 to a random value 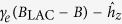. The effect of the Overhauser field is equivalent to a random magnetic field 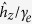 on the NV center, which makes it more difficult to tune the external magnetic field to match the resonance of the nuclear spin raising and lowering transitions. More precisely, a finite mismatch 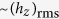 makes the originally resonant raising (lowering) transition off-resonant, and hence reduce the resonant DNP rate by a factor 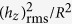. For example, a natural abundance of ^13^C nuclei gives a typical Overhauser field 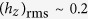 MHz. This reduces the typical DNP rate by a factor of 2 for the optical pumping rate 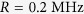, e.g., the maximal DNP rate 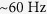 [Fig. 4(d)] for a ^13^C nucleus at 2 nm away from the NV center is reduced to 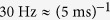, which is still sufficient to overcome the slow nuclear depolarization 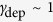 s^−1^. Therefore, under weak pumping 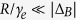, the typical Overhauser field fluctuation 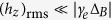 does not significantly influence the nuclear polarization.

For *N* nuclear spin-1/2’s, the number of variables 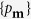 is 2^*N*^. When *N* is small, we can solve Eq. (5) exactly. For large *N*, however, the exponentially growing complexity prohibit an exact solution to Eq. (5). Here, we introduce a mean-field method (see Methods). It provides a good approximation to the average polarization 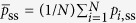 and average Overhauser field 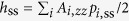, but does not include any spin-spin correlation effect, such as feedback induced spin bath narrowing^12^,^30^,^31^,^32^,^45^,^46^,^47^,^48^.

**Figure 5:**
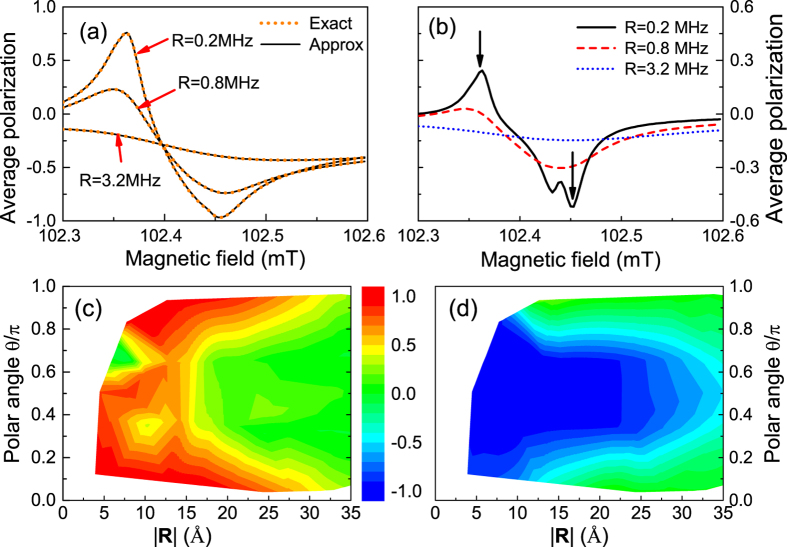
Average polarization 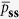 of (**a**) *N* = 7 and (**b**) *N* = 400 randomly chosen ^13^C nuclei for different optical pumping rates *R* =  0.2, 0.8, and 3.2 MHz. For *R* = 0.2 MHz, the spatial distribution of the polarization of *N* = 400 randomly chosen ^13^C nuclei in a magnetic field (**c**) 102.36 mT and (**d**) 102.45 mT [marked by the arrows in panel (b)]. The depolarization rate 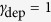 Hz for all panels.

As shown in Fig. 5(a), for a small number (*N* = 7) of randomly chosen ^13^C nuclei coupled to the NV via dipolar HFI, the average polarization 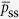 from the mean-field approximation agrees well with the exact solution to Eq. (5). For *N* = 400 randomly chosen ^13^C nuclei, the exact solution is no longer available and we plot the approximation results in Fig. 5(b). Both Figs 5(a),(b) clearly demonstrate that with decreasing optical pumping rate, the nuclear polarization shows a successively stronger peak (dip) at the resonant magnetic field 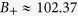 mT 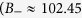 mT). The relatively stronger polarization at *B*_−_ arises from the dependence 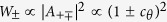 on the polar angle *θ* of the nuclear spin location: more nuclear spins have 
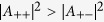
 [as plotted on top of Figs 4(a),(b)] and hence favors negative polarization. These results clearly demonstrate the possibility to strongly polarize weakly coupled ^13^C nuclear spins by using weak optical pumping near *B*_±_. The polarization of these weakly coupled ^13^C nuclei is ultimately limited by nuclear depolarization, which becomes significant when the HFI becomes too weak. This can be clearly seen in the spatial distribution of the nuclear polarization near *B*_+_ [Fig. 5(c)] and *B*_−_ [Fig. 5(d)]. Near *B*_+_, strong positive polarization is achieved for ^13^C nuclei with 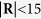 Å away from the NV center, corresponding to dipolar HFI strength >6 kHz. For more distant ^13^C nuclei, the HFI strength is too weak for the DNP to dominate over the nuclear depolarization, so their polarization drops significantly. Similarly, near *B*_−_, strong negative polarization can be achieved for ^13^C nuclei with 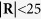 Å away from the NV center, corresponding to dipolar HFI strength ~1 kHz.

Finally, we recall that the on-site ^15^N or ^14^N nucleus has an isotropic transverse HFI and hence 
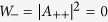
, i.e., they could be completely polarized and thus does not significantly influence the DNP of the ^13^C nuclei, except for a shift of the 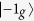 – 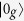 separation by ~2.2 MHz (for ^14^N) or ~3 MHz (for ^15^N), corresponding to a shift of the resonance magnetic field by ~0.78 G (for ^14^N) or ~1.1 G (for ^15^N).

## Discussion

In conclusion, we have presented a comprehensive theoretical understanding on the dynamic nuclear polarization induced by an optically pumped NV center near the ground state anticrossing at ambient temperature. Our results not only provide a clearly physical picture for a recently observed^24^ magnetic field dependence of the polarization direction of first-shell ^13^C nuclei, but also reveals an efficient scheme to strongly polarize weakly coupled ^13^C nuclear spins ~25 Å away from the NV center (HFI strength ~1 kHz) by tuning the magnetic field under weak optical pumping. These results provide a clear guidance for optimizing future dynamic nuclear polarization experiments. For example, this scheme could be used to polarize distant ^13^C nuclei in isotope purified diamond^4^ to further prolong the NV coherence time. An important limitation of our method is that it requires good alignment of the magnetic field along the N-V axis, because a tilted magnetic field would dramatically decrease the population on 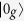 and hence degrade the nuclear polarization. For nanodiamonds containing a single NV center, we need to first align this NV center to the magnetic field before using this NV center to polarize weakly coupled ^13^C nuclear spins.

## Methods

### Derivation of nuclear spin transition rates

According to the theory^30^,^31^,^32^, the populations 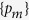 of a single nuclear spin-*I* on its Zeeman sublevels 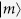 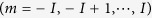 obey the rate equations


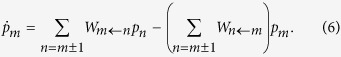


Up to second order of the *tranvserse* HFI 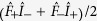 (longitudinal HFI 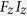 treated *exactly*), the transition rate from 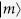 to 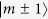 is^32^


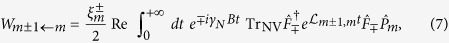


where 
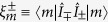
, the Liouville superoperator 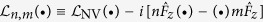, and 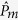 is the NV steady state in the rotating frame conditioned on the nuclear spin state being 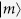, i.e., 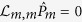 and 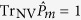.

Since we consider the NV center near the GSLAC, we can exclude the energy levels 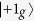 and 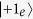, which have vanishingly small occupation. Under this approximation, the rotating frame Hamiltonian of the five-level NV model is


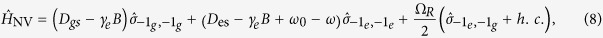


where 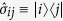. Neglecting 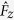-induced NV spin mixing, the longitudinal HFI reduces to 
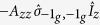
. Neglecting the non-collinear term 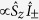 (as the NV mostly stays in 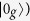 and the NV excited state HFI (which is far off-resonant), the transverse HFI reduces to 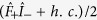 with 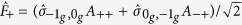. To calculate 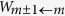 from Eq. (7), we first determine the NV steady state as 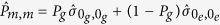, where 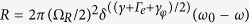 is the optical pumping rate for 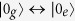. Substituting into Eq. (7) gives


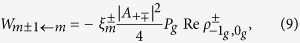


where 
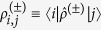
 is the 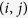 matrix element of the operator 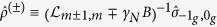, which is a linear combination of 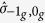, 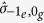, 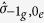, and 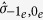.

Now we calculate 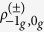 by taking the 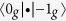, 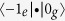, 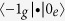 and 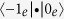 matrix elements of


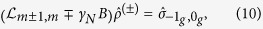


which gives four coupled equations


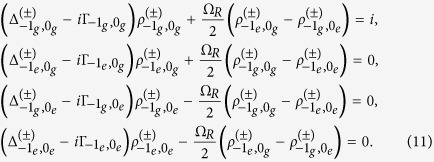


Here 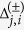 is the energy difference between 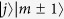 and 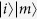 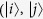 are NV states and 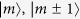 are nuclear Zeeman states), i.e.,


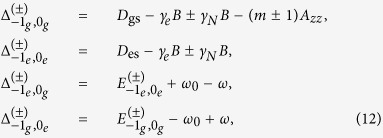


and 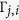 is the linewidth of the NV transition 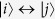, i.e., 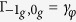, 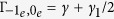, 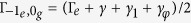, and 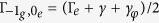. Eliminating 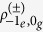 and 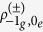 gives the “ rate equations”:


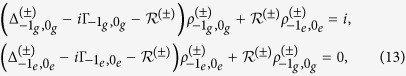


from which we obtain the solution


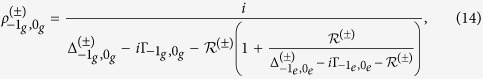


where 
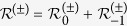
 with 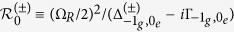 and 
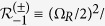
 
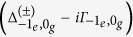
the *complex* self-energy corrections to 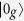 and 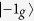 by the optical pumping 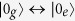 and 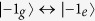, respectively. More precisely, the optical pumping 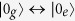 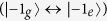 induces an optical Stark shift 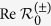 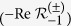 and an effective dissipation 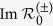 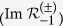 for the NV ground state 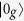 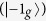. Taking 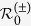 as an example, if 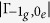 is much smaller than 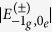, then the optical Stark shift 
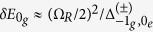
 reduces to the conventional form of a second-order energy correction in non-degenerate perturbation theroy, while the dissipation 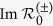 takes the semiclassical form of a Fermi golden rule. Substituting Eq. (14) into Eq. (9) immediately gives 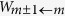, which assumes a tedious form as it includes various quantum coherence effects.

For simplification, we use the fact that the NV excited state dephasing 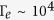 GHz 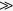 other NV dissipation 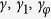 and typical detuning 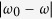, 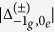, and 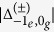, and further restrict to weak optical pumping 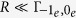 (~26.3 MHz). In this case, the optical Stark shift is negligible and the optical pumping rate simplifies to 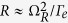, so the self-energies 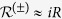 only induces NV level broadening. Substituting the resulting expression 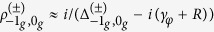 into Eq. (9) gives


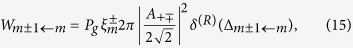


For spin-1/2, Eq. (15) simplifies to Eq. (2) of the main text.

### Mean field method for multiple nuclear spins

The essential idea of the mean-field approximation is to assume small inter-spin correlation, so that the *N*-nuclei density matrix 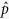 can be factorized as 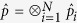, where the *i*th nuclear spin state 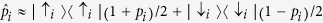 is described by its polarization 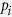. This dramatically reduces the number of variables from 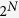 many-nuclei populations 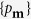 to *N* single-spin polarizations 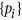. Substituting this approximation into Eq. (5) and tracing over all the nuclei except for the *i*th nucleus give *N* coupled equations:


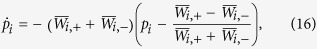


where 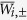 is the spin flip rate of the *i*th nucleus averaged over the states of all the other 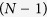 nuclear spins:


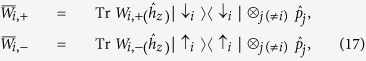


i.e., 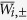 depends on the polarizations 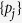 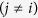 of all the other nuclear spins. Therefore, Eq. (16) with 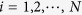 form *N* coupled differential equations for 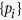. The steady-state solutions 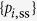 are obtained by solving *N* coupled nonlinear equations with recursive methods.

## Additional Information

**How to cite this article**: Wang, P. *et al.* Strongly polarizing weakly coupled ^13^C nuclear spins with optically pumped nitrogen-vacancy center. *Sci. Rep.* **5**, 15847; doi: 10.1038/srep15847 (2015).
